# Exercise modality influences oxygen pulse flattening during incremental cardiopulmonary exercise testing in healthy adults

**DOI:** 10.1007/s00421-026-06437-w

**Published:** 2026-09-28

**Authors:** Gur Mainzer, Faris Khourey, Merav Zucker-Toledano, Ronen Bar-Yoseph, Einat Kodesh

**Affiliations:** 1 Pediatric Cardiology unit, Padeh Medical Center, Poriya, Israel; 2 Department of Physical Therapy, Faculty of Social Welfare and Health Science, University of Haifa, Haifa, Israel; 3 Pediatric Cardiology Institute, Ruth Children’s Hospital, Rambam Health Care Campus, Haifa, Israel; 4 Pediatric Pulmonary Institute, Ruth Children’s Hospital, Rambam Health Care Campus, Haifa, Israel; 5 The Technion Faculty of Medicine, Haifa, Israel

**Keywords:** O_2_ pulse, Trajectory, Cardiac risk factor, Maximal exercise test, Ergometer, CPET

## Abstract

**Purpose:**

To compare oxygen pulse (O₂P) responses during cardiopulmonary exercise testing (CPET) in healthy adults using two ergometers, Treadmill and Cycle ergometer, and to determine the prevalence of O₂P flattening and the modality-specific characteristics of O₂P curve dynamics.

**Methods:**

Thirty-four healthy adults (29.4 ± 6.9 years; 35% female) completed two maximal CPETs on both Cycle and Treadmill, in a random order within three weeks. O₂P flattening, defined as the absence of a further increase in O₂P despite increasing workload, was assessed visually and confirmed quantitatively.

**Results:**

Twelve participants (35%) demonstrated O₂P flattening or decline, nine (27%) exclusively during Treadmill testing. A significant group × modality interaction was observed for peak O₂P (*p* < 0.05), with lower values in participants with flattening than in those showing a continuous rise. (Cycle: 9.9 ± 3.2 vs. 13.6 ± 2.7 ml/min/beat; Treadmill: 11.8 ± 3.7 vs. 16.6 ± 3.7 ml/min/beat). In addition, an O_2_P of ≤ 12.8 ml/beat on the Cycle increased the likelihood of flattening during maximal Treadmill exercise by 7.5 times (OR 7.5, *p* = 0.03).

**Conclusion:**

O₂P flattening was more frequently observed during treadmill exercise, indicating that O₂P trajectories are exercise modality dependent. In individuals with low peak O₂P during cycle ergometry, additional assessment using treadmill CPET may offer complementary information.

## Introduction

Oxygen pulse (O_2_P) defined as the amount of oxygen consumed per heartbeat, is often used as an indirect measure of cardiac performance. O_2_P is evaluated during a cardiopulmonary exercise test (CPET) and is calculated on a breath-by-breath basis as the ratio of oxygen uptake to heart rate (V̇O₂/HR). This value typically increases progressively with incremental exercise intensity (Oliveira et al. 2011). The Fick equation determines V̇O_2_ as the product of cardiac output and arteriovenous oxygen difference [(A-V)D], with the cardiac output depending on stroke volume (SV) and HR. Accordingly, O_2_P reflects the product of stroke volume and the arteriovenous oxygen difference and can be expressed as SV*(A-V) _D_ (Wasserman 1997). In healthy adults the (A-V) _D_ increases with incremental exercise whereas SV typically plateaus at moderate-to-high workloads (Higginbotham et al. 1986; Stringer et al. 1997; Turley and Wilmore 1997). Therefore, the absence of a further increase in the O₂P rise suggests a limitation in stroke volume augmentation and/or peripheral oxygen extraction (Milani et al. 2025). This relationship was demonstrated by Accalai et al. (Accalai et al. 2021), who assessed SV using an inert gas rebreathing technique. Therefore, the O_2_P response pattern during incremental exercise tests (O_2_P curve) may provide indirect information regarding the combined contributions of stroke volume and peripheral oxygen extraction. Flattening of the O₂P curve may indicate a reduced capacity for further augmentation of one or both components. This pattern has been reported in individuals with myocardial ischemia (Laukkanen et al. 2006; Accalai et al. 2021; Belardinelli et al. 2003) on one hand and in athletes (Van De Sande et al. 2019) on the other, and warrants further evaluation.

Although CPET can be performed using various ergometers, such as treadmills (TM), cycles (CE), and arm cranks, etc. (Pritchard et al. 2021), maximal exercise performed on different ergometers engages distinct physiological mechanisms due to variations in body posture, muscle mass recruitment, and hemodynamic load, resulting in mode-specific cardiorespiratory and metabolic responses, (Kodesh et al. 2024; Price et al. 2022). These variations include differences in specific reference values for exercise parameters such as modality-specific values for peak V̇O_2_ (V̇O_2_Peak), Heart Rate (HR), and Anaerobic Threshold (AT) (Cornelis and Buys 2016; Kodesh et al. 2024; Ravikiran Kisan et al. 2016). Because the O₂P response during incremental exercise provides indirect information regarding the integrated cardiovascular and peripheral responses to exercise, flattening of the O₂P curve is commonly interpreted as a potential marker of cardiovascular limitation during CPET. Determining whether this pattern occurs in healthy adults and whether it is influenced by exercise modality is therefore important for appropriate clinical interpretation. If O₂P curve dynamics differ between exercise modalities, flattening observed in one modality may reflect modality-specific physiological demands rather than a response consistent across exercise conditions, highlighting the importance of considering the ergometer used when interpreting O₂P responses.

Therefore, this study aimed to compare O₂P values and curve dynamics during maximal treadmill and cycle-ergometer CPET in healthy adults and to determine whether any observed flattening was consistent across the two modalities. We hypothesized that healthy adults without known cardiovascular disease would exhibit similar O₂P response patterns during treadmill and cycle-ergometer CPET.

## Methods

A cross sectional prospective study assessing cardiopulmonary and exercise capacities in healthy adults using CPET was conducted at a university-affiliated medical center. The study was approved by the Institutional Review Board (33-21-POR). ClinicalTrials.gov Identifier: NCT05205642.

### Participants

Thirty-four adults aged 20–40 years, with no known disease or self-reported limitation to physical exertion, as verified by the Physical Activity Readiness Questionnaire (Thomas et al. 1992), were enrolled. All participants provided written informed consent before participation. Participants were untrained, minimally active, corresponding to 600–1500 MET·min·week⁻¹ according to the International Physical Activity Questionnaire (Booth et al. 2003).

Exclusion criteria: Individuals were excluded if they had any limitation to exercise due to cardiovascular, respiratory, neurological, metabolic, or orthopedic conditions; engaged in routine exercise and more than 1500 MET·min/week of physical activity or were sedentary with less than 600 MET·min/week; had resting blood pressure above 140/90 mmHg or resting heart rate above 100 beats/min; or provided a positive response to any item on the Physical Activity Readiness Questionnaire.

### Study design

Each participant completed two CPETs, one on TM and one on CE, at the same time of day and in a random order, with both tests completed within a three-week period.

Pre-exercise evaluation included anthropometric measurements (height, weight), level of activity assessed using the International Physical Activity Questionnaire (18), and resting heart rate (HR), peripheral oxygen saturation (SpO_2_), and blood pressure (BP). Spirometry was conducted in accordance with the American Thoracic Society/European Respiratory Society guidelines (Graham et al. 2019) to assess respiratory volume, flow, and maximal voluntary ventilation (MVV), using a Quark PFT spirometer (COSMED, Italy). This spirometry examination was repeated ten minutes post exercise to rule out exercise-induced bronchoconstriction, defined as a decrease of > 12% from pre-exercise FEV_1_.

Each participant underwent a maximal incremental exercise test using the Ramp test. The CE Workload was increased by 15 W·min⁻¹ in females and 20 W·min⁻¹ in males, and the Ramped Bruce Protocol was performed on TM until exhaustion.

The following cardiopulmonary parameters were recorded: HR from 12-lead ECG, Blood Pressure (BP), respiration rate (RR), SpO_2_, tidal volume (TV), minute ventilation (VE), breathing reserve (BR), oxygen uptake (VO_2_), carbon dioxide production (VCO_2_), end-tidal carbon dioxide (ETCO_2_), respiratory equivalents (VE/VO_2_, VE/VCO_2_), VO_2_ at the anaerobic threshold (AT), O_2_P, and respiratory exchange ratio (RER).

The following indices were calculated to assess myocardial function: (1) Rate Pressure Product (RPP) peak systolic blood pressure multiplied by peak heart rate (HR), as an index of myocardial oxygen demand; (2) Circulatory Power, as peak systolic blood pressure multiplied by peak V̇O₂; (3) Ventilatory Power, as peak systolic blood pressure divided by the V̇E/V̇CO₂ slope; and (4) oxygen uptake efficiency slope (OUES), as the relationship between V̇O₂ and log₁₀ V̇E.

Maximal effort was defined by a V̇O₂ plateau (failure of oxygen uptake to increase despite increasing work) or achievement of at least two of the following: RER > 1.15, maximal HR > 90% of age-predicted maximal HR (Machado and Denadai 2011; Mahon et al. 2010), or rating of perceived exertion ≥ 8 on the 0–10 Borg scale.

**Fig. 1:**
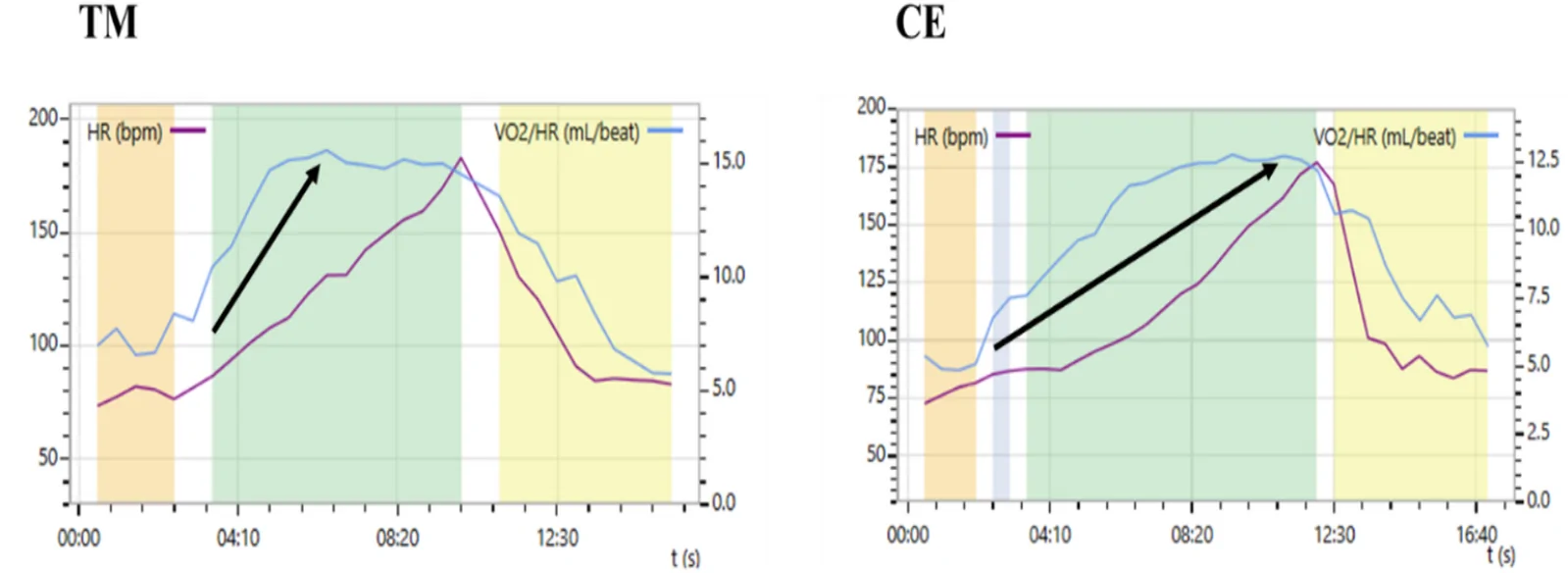
Examples of O_2_P Flattening Curves.: The left panel shows an example of an O_2_P plateau on a treadmill ergometer (the black arrow indicates the linear increase until the plateau). The right panel demonstrates a typical O_2_P increase during cycle ergometer exercise. The purple line represents heart rate, and the blue line represents O_2_P

O₂P flattening was defined as the absence of a further increase of the V̇O₂/HR curve despite simultaneous increases in workload and V̇O₂. The flattening point was the first point at which the curve stopped increasing (Fig. 1). O₂P trajectory was initially classified by visual inspection (Mapelli et al. 2022) and subsequently confirmed from the breath-by-breath values. The first ventilatory threshold was identified from the gas-exchange response using concordance between the V-slope and ventilatory-equivalent methods. (Dores et al. 2024) Participants were categorized as: (1) O₂P-F_DUAL_, flattening on both TM and CE; (2) O₂P-F_TM_, flattening on TM only; or (3) O₂P-N, no flattening on either ergometer.

### Statistical analysis

Continuous variables are presented as mean ± SD or median and range, as appropriate, and categorical variables as frequencies and percentages. Because the O₂P-F_DUAL_ group comprised only three participants, inferential analyses were limited to the O₂P-F_TM_ and O₂P-N groups. Baseline characteristics variables were compared between groups using independent-samples t tests. CPET-derived outcomes were analyzed using linear mixed-effects models with modality, group, and their interaction as fixed effects and an unstructured covariance matrix for repeated measures. Model-based contrasts were used for between-group and within-group comparisons. Peak O₂P, peak V̇O₂, and peak HR were considered a single physiological outcome family; Holm adjustment was applied separately to the between-group, within-group, and interaction tests. All other outcomes were analyzed exploratorily using unadjusted p values. Logistic regression was used to assess the association between peak O₂P during CE and O₂P flattening during TM. All tests were two-sided, with statistical significance set at *p* < 0.05. Analyses were performed using SAS version 9.4 for Windows.

## Results

Thirty-four healthy adults aged 29.4 ± 6.9 years (12 females; 35%) completed two maximal CPETs on TM and CE ergometers within three weeks. The participants’ International Physical Activity Questionnaire score was 967 ± 216 METs/week, indicating a moderate physical activity level.

Twenty-two participants had a continuously increasing O₂P pattern. Twelve (35%) demonstrated O₂P flattening or decline during CPET: three on both TM and CE (O₂P-F_DUAL)_ and nine on TM only (O₂P-FTM), with a continuously increasing trajectory during CE (Fig. 1).

Table 1 presents the anthropometric, demographic, and physical activity levels of the three groups. No statistically significant differences were found for these variables between the O2P-FTM and O2P-N groups, the two groups focused on in this study.

**Table 1:** Baseline characteristics of groups classified by O₂P pattern

	O_2_P-F_DUAL_	O_2_P-F_TM_	O_2_P-*N*	p-value *
N (% of participants)	3 (8.8%)	9 (26.5%)	22 (64.7%)	
Female [n (%)]	1 (33%)	5 (56%)	6 (27%)	0.14
Age [years]	30.7 ± 9.0	28.2 ± 6.0	29.7 ± 7.2	0.59
BMI [kg/m^2^]	24.7 ± 5.5	26.8 ± 6.6	29.3 ± 6.7	0.36
IPAQ [METs min/week]	1037 ± 142	881 ± 198	1064 ± 210	0.28

*Between O_2_P-F_TM_ and O_2_P-N groups

*BMI* Body Mass Index, *IPAQ* International Physical Activity Questionnaire, O_2_P-F_DUAL_ – Oxygen Pulse flattening on both TM and CE, *O*_*2*_*P-F*_*TM*_ Oxygen Pulse flattening in TM only, *O*_*2*_*P-N* no Oxygen Pulse flattening

CPET-derived outcomes by group and exercise modality are presented in Table 2.

Resting V̇O₂, HR, and peak systolic blood pressure did not differ significantly between the O₂P-F_TM_ and O₂P-N groups in either modality. Within both groups, peak V̇O₂, peak O₂P, and peak HR were higher during TM than CE. For peak O₂P, however, the magnitude of the modality-related increase differed between groups: the increase from CE to TM was smaller in the O₂P-FTM group than in the O₂P-N group, resulting in a significant group × modality interaction (*p* = 0.044). Between-group comparisons showed lower peak O₂P in the O₂P-F_TM_ group during TM, whereas the difference during CE did not reach statistical significance. Peak V̇O₂ and peak HR showed no significant between-group differences in either modality and no significant group × modality interactions. Peak HR, expressed as a percentage of age-predicted maximal HR, was also higher during TM than during CE within both groups, with no significant between-group differences or interaction. Among the exploratory outcomes, RER showed a significant group × modality interaction (*p* = 0.021), indicating a higher RER during TM than during CE in the O₂P-N group, but not in the O₂P-FTM group. OUES was lower in the O₂P-F_TM_ group than in the O₂P-N group in both modalities, without a significant group × modality interaction.

**Table 2:** CPET-derived outcomes by group and exercise modality

Outcome	O₂*P*-F_TM_ mean ± SD CE / TM	O₂*P*-*N* mean ± SD CE / TM	Between groups *p*	Within group *p*	Group × Modality Interaction *p*		
***Rest***							
V̇O₂, mL·kg⁻¹·min⁻¹	CE: 4.58 ± 0.82 TM: 4.98 ± 1.01	CE: 4.85 ± 1.39 TM: 4.87 ± 0.95	CE: 0.506 TM: 0.793	O₂P-F_TM_: 0.254 O₂P-N: 0.933		0.381	
HR, beats·min⁻¹	CE: 88.00 ± 11.28 TM: 86.00 ± 9.90	CE: 79.41 ± 10.46 TM: 80.32 ± 11.19	CE: 0.070 TM: 0.181	O₂P-F_TM_: 0.608 O₂P-N: 0.700		0.520	
SBP mmHg	CE: 116.67 ± 5.00 TM: 117.78 ± 10.03	CE: 116.14 ± 10.46 TM: 119.32 ± 6.78	CE: 0.850 TM: 0.681	O₂P-F_TM_: 0.622 O₂P-N: 0.134		0.494	
**Peak Exercise variables**							
Power, W	CE: 140.56 ± 59.58	CE: 189.09 ± 45.90					
Speed, km·h⁻¹	TM: 5.83 ± 1.23	TM: 6.35 ± 0.85					
Grade, %	TM: 14.22 ± 1.64	TM: 14.91 ± 1.31					
Peak O₂Pᵃ, mL·min⁻¹·beat⁻¹	CE: 9.93 ± 3.20 TM: 11.78 ± 3.66	CE: 13.56 ± 2.67 TM: 16.59 ± 3.67	CE: 0.051 **TM: 0.028**	O₂P-F_TM_: **<0.001** O₂P-N: **<0.001**		**0.044**	
Peak V̇O₂ᵃ, mL·kg⁻¹·min⁻¹	CE: 23.21 ± 7.39 TM: 29.01 ± 7.61	CE: 26.69 ± 6.86 TM: 34.85 ± 7.68	CE: 0.734 TM: 0.290	O₂P-F_TM_: <**0.001** O₂P-N: <**0.001**		0.107	
Peak HRᵃ, beats·min⁻¹	CE: 171.11 ± 13.21 TM: 183.89 ± 12.43	CE: 169.27 ± 20.19 TM: 181.09 ± 14.05	CE: 1.000 TM: 1.000	O₂P-F_TM_: **0.013** O₂P-N: <**0.001**		0.840	
Peak HR, % predicted	CE: 90.9 ± 7.2 TM: 97.8 ± 8.1	CE: 90.3 ± 9.7 TM: 96.7 ± 5.9	CE: 0.871 TM: 0.667	O₂P-FTM:**0.002** O₂P-N: <**0.001**		0.826	
Peak SBP mmHg	CE: 159.44 ± 24.80 TM: 170.00 ± 18.71	CE: 177.05 ± 19.62 TM: 180.91 ± 17.16	CE: 0.081 TM: 0.154	O₂P-F_TM_: 0.164 O₂P-N: 0.286		0.404	
RER	CE: 1.17 ± 0.05 TM: 1.19 ± 0.09	CE: 1.18 ± 0.08 TM: 1.28 ± 0.10	CE: 0.516 TM: 0.033	O₂P-F_TM_: 0.255 O₂P-N: **<0.001**		**0.021**	
**Cardiovascular indexes**							
Circulatory power	CE: 3,807 ± 1,675 TM: 5,008 ± 1,690	CE: 4,757 ± 1,467 TM: 6,362 ± 1,745	CE: 0.161 TM: 0.060	O₂P-F_TM_: **0.002** O₂P-N: **<0.001**		0.242	
Ventilatory power	CE: 6.32 ± 1.84 TM: 6.95 ± 1.48	CE: 7.19 ± 1.66 TM: 7.45 ± 1.43	CE: 0.239 TM: 0.398	O₂P-F_TM_: 0.145 O₂P-N: 0.256		0.427	
RPP	CE: 27,519 ± 6,127 TM: 31,314 ± 4,473	CE: 30,230 ± 6,171 TM: 32,819 ± 4,443	CE: 0.282 TM: 0.408	O₂P-F_TM_: 0.046 O₂P-N: 0.006		0.519	
**Sub-Maximal parameters**							
V̇O₂@AT mL·kg⁻¹·min⁻¹	CE: 16.42 ± 3.43 TM: 23.41 ± 5.73	CE: 17.40 ± 4.96 TM: 25.84 ± 6.63	CE: 0.538 TM: 0.321	O₂P-F_TM_: **<0.001** O₂P-N: **<0.001**		0.306	
V̇E/V̇CO₂ slope	CE: 26.24 ± 4.21 TM: 25.08 ± 3.90	CE: 25.35 ± 3.83 TM: 24.80 ± 3.39	CE: 0.588 TM: 0.852	O₂P-F_TM_: 0.278 O₂P-N: 0.447		0.622	
V̇E/V̇CO₂ intercept	CE: 5.91 ± 1.82 TM: 7.07 ± 2.48	CE: 6.15 ± 2.63 TM: 6.43 ± 3.35	CE: 0.775 TM: 0.567	O₂P-F_TM_: 0.300 O₂P-N: 0.632		0.477	
OUES	CE: 1,907 ± 532 TM: 2,112 ± 792	CE: 2,468 ± 523 TM: 2,922 ± 490	**CE: 0.017** **TM: 0.016**	O₂P-F_TM_: 0.183 **O₂P-N: <0.001**		0.158	

Data are presented as mean ± SD. Between-group *p* values compare O₂P-F_TM_ with O₂P-N separately within CE and TM. Within-group *p* values compare CE with TM separately within O₂P-F_TM_ and O₂P-N. The group × modality interaction tests whether the difference between exercise modalities differs between groups

ᵃHolm adjustment was applied separately to the between-group, within-group, and group × modality comparisons for peak O₂P, peak V̇O₂, and peak HR

All other p values are unadjusted and should be interpreted as exploratory

AT, anaerobic threshold; CE, cycle ergometry; HR, heart rate; O₂P, oxygen pulse; O₂P-FTM, treadmill-only O₂P flattening; O₂P-N, no O₂P flattening; OUES, oxygen uptake efficiency slope; RER, respiratory exchange ratio; RPP, rate pressure product; SBP, systolic blood pressure; TM, treadmill; V̇CO₂, carbon dioxide output; V̇E, minute ventilation; V̇O₂, oxygen uptake.

When flattening of the O_2_P curve was detected during exercise on TM, the time of flattening and the corresponding V̇O_2_, HR, and O_2_P values were recorded (see Table 3). Eleven participants (92%) experienced O_2_P flattening on the treadmill before the 1st ventilatory anaerobic threshold.

**Table 3:** Flattening values and timing on the Treadmill exercise

Group	Participant #	O_2_P Curve pattern	Time to O_2_P Flattening	Time to VT	Time to Peak	*Values at flattening point*		
						**HR** (bpm)	**V̇O**_**2**_ (ml/kg/min)	**O**_**2**_ **P**
O_2_P-F_TM_	1	Flattening	08:03*	09:05	15:10	133	25.7	14.5
	2	Flattening	04:17*	06:16	09:43	158	22.0	7.7
	3	Flattening	05:44*	08:13	12:08	129	19.8	8.5
	4	Flattening	06:08*	10:01	14:36	113	22.4	16.6
	5	Descending	06:48	06:40	09:16	154	22.1	8.1
	6	Flattening	07:28*	11:01	12:47	111	25.7	15.3
	7	Flattening	05:37*	07:05	09:20	139	17.8	11.8
	8	Descending	05:35*	07:16	09:56	119	18.0	15.7
	9	Flattening	04:52*	05:32	07:23	124	15.4	10.1
O_2_P-F_DUAL_	10	Descending	05:48*	07:55	11:03	104	21.5	11.8
	11	Flattening	06:51*	07:35	11:44	124	20.0	16.5
	12	Flattening	09:00*	09:31	13:53	119	23.8	14.6

* O_2_P flattening before the ventilatory anaerobic threshold

*VT* First Ventilatory Threshold, *HR* Heart Rate, *O*_*2*_*P* Oxygen Pulse, *O*_*2*_*P-F*_*TM*_ O_2_P Oxygen Pulse flattening in TM only, *O*_*2*_*P-F*_*DUAL*_ Oxygen Pulse flattening on both TM and CE, *V̇O*_*2*_ Oxygen uptake

The TM exercise specific values of V̇O₂, HR, and O_2_P at the identified flattening points were subsequently determined and matched to the corresponding time points during the CE exercise. The timing of these matched values on the cycle ergometer is presented in Table 4.

All nine individuals in the O_2_P-FTM group reached the HR value associated with flattening on the TM, seven participants (78%) reached the corresponding V̇O₂, value, and only one participant (11%) reached the equivalent O_2_P value occurring near the end of the test within ~ 40 s of peak exercise.

**Table 4:** Values and Timing on Cycle ergometer

Group	Participant #	O_2_P Curve pattern	Time to O_2_P Flattening	Time to VT	Time to Peak	Time during CE for achieving *TM flattening values*		
						HR	V̇O₂,	O_2_P
O_2_P-F_TM_	1	Normal	No	08:21	12:52	08:29	09:49	12:12
	2	Normal	No	06:11	08:16	07:22	**NR**	**NR**
	3	Normal	No	06:49	08:30	06:07	08:08	**NR**
	4	Normal	No	08:44	14:38	07:16	10:50	**NR**
	5	Normal	No	06:31	08:36	06:54	**NR**	**NR**
	6	Normal	No	07:47	12:00	05:09	07:41	**NR**
	7	Normal	No	06:52	10:04	08:06	09:58	**NR**
	8	Normal	No	08:01	11:44	07:42	09:37	**NR**
	9	Normal	No	06:47	09:02	06:31	08:20	**NR**
O_2_P-F_DUAL_	10	Descending	08:23	06:28	09:43	06:37	09:08	**NR**
	11	Flattening	13:37	08:36	14:09	08:48	10:42	13:37
	12	Flattening	07:40*	08:12	12:16	08:07	08:05	**NR**

* O_2_P flattening before the anaerobic threshold

*AT* Anaerobic Threshold, *HR* Heart Rate, *NR* Not Reached, *O*_*2*_*P* Oxygen Pulse, *O*_*2*_*P-F*_*TM*_ O_2_P Oxygen Pulse flattening in TM only, *O*_*2*_*P-F*_*DUAL*_ Oxygen Pulse flattening on both TM and CE, *V̇O*_*2*_ Oxygen uptake

**Fig. 2:**
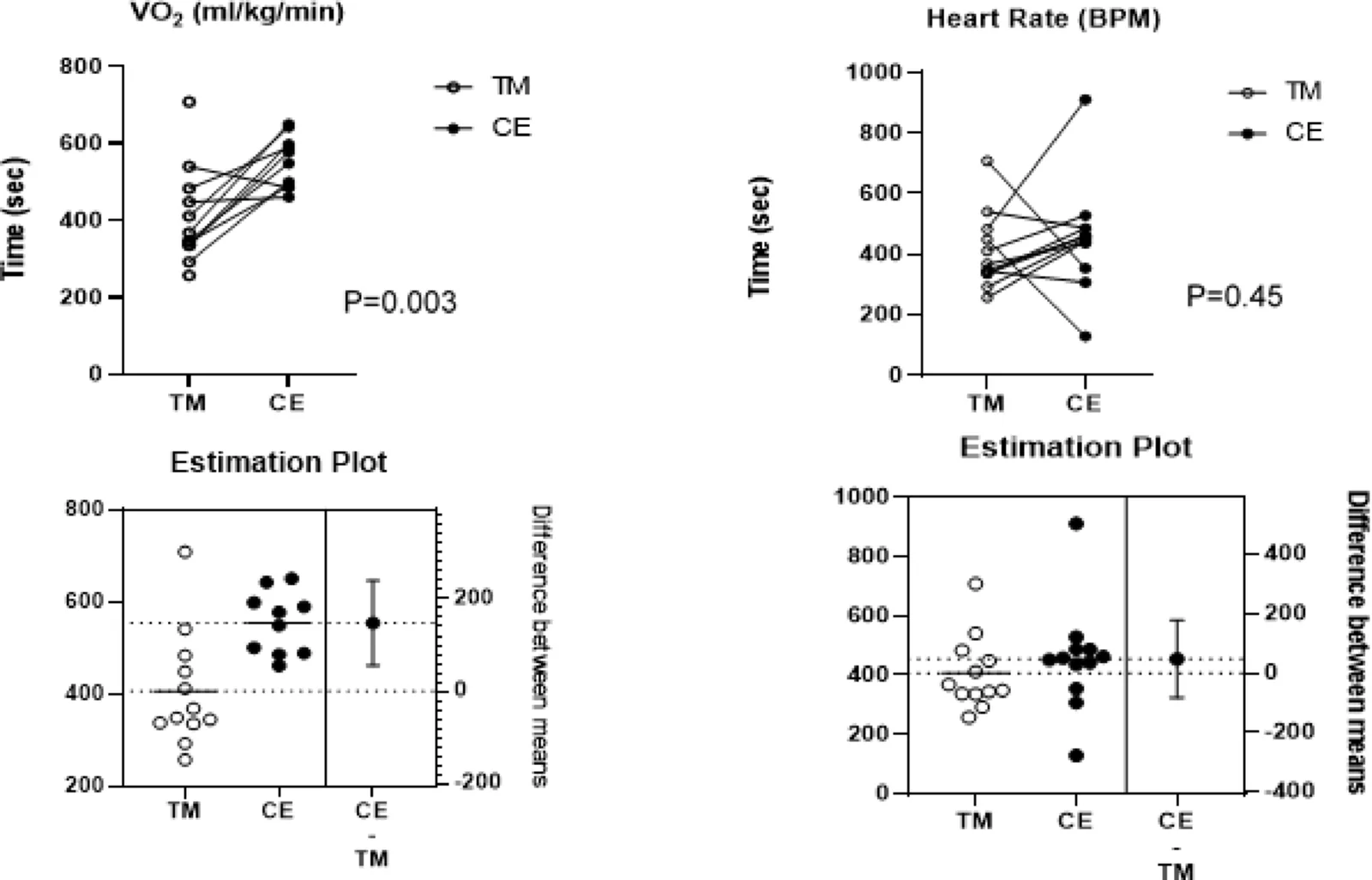
Time points at which participants reached the V̇O₂ **A** and HR **B** values corresponding to O₂P flattening during treadmill (TM) exercise, and the matching values obtained during the cycle ergometer (CE) test. The V̇O₂ value was reached significantly later on the CE, whereas the HR time point did not differ between modalities at the O₂P flattening point

A comparison of the time points at which participants reach O_2_P flattening and the corresponding V̇O₂, and HR values in both TM and CE exercises (Fig. 2) revealed modality-specific differences. The time points corresponding to the V̇O₂, values associated with O_2_P flattening occurred significantly later on the CE compared to TM (Fig. 2A). In contrast, the time required to reach the HR values at O_2_P flattening did not differ significantly between the two ergometers (Fig. 2B).

The logistic regression findings for predicting flattening during TM, based on the O_2_P peak value from CE, suggest that the probability of flattening increases with decreasing O_2_P peak (OR = 0.65, 95% CI [0.47, 0.91], *p* = 0.01). Additionally, when the O_2_P peak on CE is ≤ 12.8 ml/beat, the likelihood of flattening during maximal exercise on TM increases by 7.5 times (OR = 7.5, *p* = 0.03).

## Discussion

In this study of healthy, untrained adults, 12 participants (35%) demonstrated O₂P flattening or decline during maximal CPET. Only three showed this pattern in both TM and CE; the remaining nine showed it only on TM. Eight of these nine did not reach the O₂P value corresponding to their TM flattening point during CE, and one reached it only near the end of CPET with CE. Participants in the O₂P-F_TM_ group had lower peak O₂P values than those in the O₂P-N group across both exercise modalities, and the significant group × modality interaction indicated that the increase in peak O₂P from CE to TM was smaller in the O₂P-F_TM_ group than in the O₂P-N group. Peak HR and peak V̇O₂ were higher during TM than CE in both groups; however, neither variable differed significantly between groups nor showed a significant group × modality interaction. In the exploratory logistic regression, CE peak O₂*P* ≤ 12.8 ml·beat⁻¹ was associated with 7.5-fold higher odds of flattening during TM.

We found that approximately one-third of this apparently healthy cohort demonstrated O₂P flattening. Because O₂P reflects the combined contributions of stroke volume and peripheral oxygen extraction, O₂P flattening may reflect limited stroke-volume augmentation, reduced peripheral oxygen extraction, or both, and therefore cannot, by itself, be interpreted as evidence of cardiovascular dysfunction. (Guazzi et al. 2012). The O_2_P curve, which reflects the combined contributions of SV and the (A-V)_D_, typically rises progressively during exercise (Oliveira et al. 2011). Many studies report a plateau in SV at 40–50% of maximal exercise capacity in healthy subjects on both TM (Chapman et al. 1960) and CE (Higginbotham et al. 1986). In contrast, the (A-V)_D_ in a healthy population increases linearly until peak V̇O₂, is achieved (Higginbotham et al. 1986; Stringer et al. 1997; Turley and Wilmore 1997). This suggests that when SV plateaus, O_2_P is mainly determined by the A-V difference (Whipp et al. 1996). Thus, flattening or decline in the O_2_P curve could suggest impaired SV augmentation (Guazzi et al. 2012) or impaired (A-V) _D_ (Ghantous et al. 2023). In patients with coronary artery disease, a flattening or downward O_2_P curve observed during CE has been suggested to indicate the presence of myocardial fibrosis (De Lorenzo et al. 2017) and exercise-induced myocardial ischemia, as assessed by myocardial perfusion imaging (Belardinelli et al. 2003).

A similar pattern of the O_2_P curve was found in individuals with pulmonary arterial hypertension, who are characterized by impaired hemodynamics and reduced exercise SV (Degani-Costa et al. 2019).

In healthy individuals, a gradual, curvilinear rise in O_2_P with exercise intensity, reaching approximately threefold over the resting level, is anticipated (Wasserman K 2005). A low maximal O_2_P may reflect low fitness; however, the pattern of O_2_P increase is expected to be normal (Rowland et al. 2011). In our cohort, all participants were untrained and minimally active, as identified using the International Physical Activity Questionnaire, with self-reported physical activity levels of 600–1500 MET·min·week⁻¹. No significant between-group differences were found in peak V̇O₂. Therefore, the observed differences in O₂P values and response patterns were not accompanied by a significant between-group difference in aerobic capacity. Importantly, the smaller increase in peak O₂P from CE to TM in the O₂P-F_TM_ group occurred despite comparable modality-related increases in peak V̇O₂ and peak HR in both groups. The absence of corresponding group × modality interactions for peak V̇O₂ and peak HR suggests that the differential O₂P response was not simply attributable to differences in aerobic capacity or heart-rate response.

O₂P flattening has also been described in athletic populations. In one study, approximately 20% of elite soccer players demonstrated O₂P flattening during the final 2 min of maximal treadmill exercise (Perim et al. 2011). In another study, physically fit patients referred for evaluation of suspected coronary artery disease, with a mean peak V̇O₂ of 119 ± 34% of predicted, underwent clinically indicated CPET on either TM or CE within 90 days of coronary angiography. Peak O₂P was similar in those tested on CE and TM (16.7 and 18.0 mL·beat⁻¹, respectively), and approximately one-third demonstrated an O₂P plateau or decline during late exercise. This pattern showed poor diagnostic accuracy for obstructive coronary artery disease, suggesting that late-exercise O₂P flattening in physically fit individuals is not a specific marker of coronary obstruction (Petek et al. 2021). Our findings extend these observations by demonstrating that atypical O₂P patterns are not confined to physically fit populations but may also be observed in apparently healthy individuals with low habitual physical activity. Accordingly, a relatively high prevalence of atypical O₂P trajectories was evident in our cohort of healthy, untrained participants.

Other studies have reported that a mid- to late-exercise O₂P plateau after the ventilatory threshold may lie within the spectrum of normal responses (Husaini and Emery 2024; Petek et al. 2021; Vella and Robergs 2005). In the present study, however, 11 of the 12 participants with flattening on TM reached the flattening point before the first ventilatory threshold. The appearance of O₂P flattening before the first ventilatory threshold is less readily explained by the normal SV plateau typically observed during moderate-to-high exercise intensities. Because O₂P represents the product of SV and arteriovenous oxygen difference, an early plateau indicates that heart rate increased disproportionately relative to V̇O₂ and may reflect an early limitation in stroke-volume augmentation, peripheral oxygen extraction, or both. In untrained individuals, limited plasma volume expansion and lower cardiovascular conditioning may constrain stroke-volume reserve, whereas the greater active muscle mass and cardiovascular demand of treadmill exercise may make this limitation more apparent earlier. Early flattening has also been reported in 36.8% of asymptomatic adults and was associated with physical inactivity and other cardiovascular risk factors, suggesting that it may represent a relatively common subclinical physiological phenotype rather than evidence of established cardiovascular disease (de Almeida et al. 2022). In the present study, the lower peak O₂P during TM and lower OUES in both modalities in the O₂P-F_TM_ group may indicate less efficient integrated oxygen delivery and utilization. However, the OUES findings were exploratory, and because stroke volume and arteriovenous oxygen difference were not measured directly, the physiological basis of these findings cannot be determined.

Further examination showed that eight of the nine participants with flattening only during TM did not attain during CE the O₂P value observed at the TM flattening point. Nevertheless, all nine participants reached the HR associated with the TM flattening point during CE, and seven reached the corresponding V̇O₂ (Tables 3 and 4; Fig. 2), although this occurred significantly later than during TM and in most cases, after the first ventilatory threshold. Because flattening occurred well before peak exercise, the higher peak V̇O₂ achieved during TM cannot alone explain this pattern. Given that O₂P is calculated as the ratio of V̇O₂ to HR, these findings suggest that the temporal relationship between V̇O₂ and HR throughout exercise may differ between modalities: during TM, V̇O₂ no longer increased sufficiently relative to HR, whereas during CE, O₂P remained lower but continued to rise.

Previous work has shown that V̇O₂ at a given relative HR may differ between treadmill and cycle exercise, potentially because of modality-related differences in SV, arteriovenous oxygen difference, peripheral resistance, or end-diastolic volume (Olsson et al. 2022). Such mechanisms may have contributed to the different O₂P trajectories observed in the present study; however, this interpretation remains speculative because SV and peripheral oxygen extraction were not measured directly.

TM exercise generally recruits a larger active muscle mass and elicits a higher peak V̇O₂ than CE in individuals who are not specifically trained in cycling, whereas the greater local muscular demands of cycling may limit performance before maximal cardiorespiratory capacity is attained (Millet et al. 2009). Together, the findings demonstrate that O₂P trajectories in apparently healthy, untrained adults may differ according to exercise modality, even when comparable HR and V̇O₂ values are attained. This difference may reflect the combined effects of muscle recruitment, exercise familiarity, and individual cardiovascular and peripheral responses. The ergometer used should therefore be considered when interpreting O₂P flattening during CPET. At the same time, O₂P flattening during TM should not be interpreted as a stand-alone indicator of cardiovascular dysfunction, because O₂P reflects both SV and peripheral oxygen extraction, neither of which was measured directly in the present study.

Peak O₂P has prognostic value in clinical populations. In middle-aged men, peak O₂P was inversely related to mortality. Specifically, men with low O_2_P (< 13.5 ml/beat) had a 2.5 times higher risk of coronary heart disease than those with high O_2_P values (> 17.8 ml/beat) (Laukkanen et al. 2006). In the present study, CE peak O₂*P* ≤ 12.8 ml·beat⁻¹ was associated with higher odds of flattening during TM. However, given the small sample size and the exploratory nature of this threshold, this finding should be interpreted cautiously and requires confirmation in larger cohorts.

This study has several limitations. The sample was small, particularly the O₂P-FDUAL group, and the multiple exploratory comparisons increase the possibility of chance findings. Participants were screened using a self-reported health declaration, spirometry, resting vital signs, vital-sign monitoring during exercise, and 12-lead ECG recordings obtained at rest and throughout the exercise test; however, the presence of previously undiagnosed medical conditions cannot be completely excluded. Echocardiography, hemoglobin concentration, direct stroke-volume assessment, and measures of peripheral oxygen extraction were not available, limiting mechanistic interpretation. The present findings were obtained in untrained and “minimally active” adults and may not generalize to fitter or cycle-trained populations, in whom CE may elicit a greater cardiorespiratory response. Although participants with O₂P flattening were referred to their physicians, clinical and long-term follow-up data were unavailable.

## Conclusions

O₂P flattening was identified in 35% of the apparently healthy, untrained cohort and occurred more frequently during TM than CE. Overall, these findings suggest that the O₂P response observed during CPET may be influenced by exercise modality. In untrained individuals, local muscular fatigue, discomfort, or limited cycling familiarity may constrain performance during CE; however, these factors cannot fully explain the different O₂P trajectories, because the HR and V̇O₂ values associated with treadmill flattening were generally still reached during cycling. The different O₂P response may therefore reflect a modality-specific relationship between V̇O₂ and HR. The relatively high prevalence of O₂P flattening suggests that this response is not necessarily pathological and should not be interpreted in isolation as evidence of cardiovascular dysfunction. Because TM revealed non-increasing O₂P responses more frequently, it may provide complementary information when evaluating O₂P trajectories in healthy, untrained adults, particularly when peak O₂P during CE is low.

Larger studies incorporating direct measurements of stroke volume and peripheral oxygen extraction are needed to clarify the underlying mechanisms and clinical significance of O₂P flattening.

## Abbreviations

[A-V] D: Arteriovenous oxygen difference
AT: Anaerobic threshold
BMI: Body mass index
BP: Blood pressure
CE: Cycle ergometer
CPET: Cardiopulmonary exercise testing
HR: Heart rate
MVV: Maximal voluntary ventilation
O₂P: Oxygen pulse
O₂P: F_DUAL_ Group–Oxygen pulse flattening on both treadmill and cycle ergometer
O₂P: F_TM_ Group–Oxygen pulse flattening on treadmill only
O₂P: N Group–No oxygen pulse flattening
OUES: Oxygen uptake efficiency slope
RER: Respiratory exchange ratio
RR: Respiratory rate
SV: Stroke volume
TM: Treadmill
V̇CO₂: Carbon dioxide production
V̇E: Minute ventilation
V̇O₂: Oxygen uptake
V̇O₂@AT: Oxygen uptake at anaerobic threshold
VE/V̇CO₂: Ventilatory equivalent for carbon dioxide
VE/V̇O₂: Ventilatory equivalent for oxygen
VT: Ventilatory threshold
